# Editorial for the Special Issue on Droplet Microfluidics

**DOI:** 10.3390/mi11121086

**Published:** 2020-12-08

**Authors:** Eric Brouzes, Siran Li

**Affiliations:** 1 Department of Biomedical Engineering, Stony Brook University, Stony Brook, NY 11794, USA; 2 Laufer Center for Physical and Quantitative Biology, Stony Brook University, Stony Brook, NY 11794, USA; 3 Cancer Center, Stony Brook School of Medicine, Stony Brook, NY 11794, USA; 4 Institute for Engineering Driven Medicine, Stony Brook University, Stony Brook, NY 11794, USA; 5 Cold Spring Harbor Laboratories, Cold Spring Harbor, NY 11724, USA

Emulsions, which are collections of immiscible droplets, have elicited scientific and commercial interests for decades. However, droplet microfluidics has only recently emerged due to advances in microfluidics and physical chemistry. Droplet microfluidics uniquely allows encapsulation into monodisperse and stable droplets. Those properties represent the two pillars of the technology because they enable manipulating droplets as independent micro-reactors both on-chip and off-chip. Droplets are generated and processed with high precision in microfluidic circuits that can include different modules in series.

Since its inception, droplet microfluidics has inspired numerous research topics, ranging from fluid hydrodynamics to biology, chemistry, and material sciences. The field is indeed highly interdisciplinary as it combines fluid dynamics, microfabrication, physical-chemistry, chemistry, and biology. Droplet microfluidics has also garnered commercial success through applications such as digital PCR or single-cell genomics.

This special issue is a collection of eight articles that perfectly illustrate the breadth of this vibrant field. It represents a testimony to the creativity involved in the development of droplet microfluidics. Six articles cover automation and templating capabilities of droplet microfluidics for biological, chemical, and material sciences applications; the last two articles present novel modules that proposes a solution to the limited mixing in single phase microfluidics and that provides new sorting abilities for biological applications. 

Droplet microfluidics promises workflow automation with improved throughput and reduced reagent consumption. The paper by Lindong Weng et al. [1] reviews the advantages of droplet microfluidics to screen large libraries for directed evolution. Directed evolution is arguably the initial impetus behind the development of droplet microfluidics because droplets readily link the genotype (coding sequence) to the genotype (protein) via encapsulation. The paper also provides a thorough review of the microfluidic modules used to manipulate and process droplets at high throughput and thus serves as a perfect introduction to the technology. Mark Davies et al. [2] present a droplet-based commercial system’s theory and practice to generate combinatorial drug libraries for high throughput screening. The combinatorial power of droplets combined with reagent consumption reduction surpasses conventional technologies such as robotics for large and complex screening strategies. Hoon Suk Rho et al. [3] introduce a system that combines droplet microfluidics with on-chip valves, two technologies that have been recently combined together. Their low throughput platform enables fine droplet control and the design of complex chemical and biological reactions. Finally, Nan Shi et al. [4] demonstrate a screening platform with automated calibration that provides real-time correction for fluorescence drift. The repeated calibration is essential to improve the quality of analytical assays, especially those that rely on expensive reagents.

Microfluidics droplets can be used as templates for manufacturing original microscale objects. For instance, Jianhua Guo et al. [5] harness osmotic pressure to precisely tune the thickness of the ultrathin shell of microparticles manufactured at high throughput. Employing unusual fluids, Qingming Hu et al. [6] demonstrate the generation of liquid metal droplets numerically and experimentally. These two papers exemplify the ability to use droplet microfluidics to create new materials with novel properties and applications in very diverse fields.

The approach of Xiaoyu Jia et al. [7] contrasts with the typical use of droplets as microreactors by utilizing them as actuators. They overcome the limited mixing of single-phase microfluidics by creating chaotic convective turbulent flow with air bubbles. Finally, Chandler Dobso et al. [8] use a photo-reactive surfactant to tag droplets of interest that can then be passively sorted out due to the induced change in interfacial energy. Unlike conventional sorting methods, where the sorting decision is made just before sorting, this functionality temporaly uncouples observation, decision algorithm, and sorting. This uncoupling opens up the ability of long term observation of droplets before sorting.

We are grateful to all the authors who submitted their papers to this special issue dedicated to droplet microfluidics. We are also indebted to all the reviewers who dedicated their time and helped improve the quality of the submitted papers.
